# Death Following Vaccination

**Published:** 1848

**Authors:** P. Gregg

**Affiliations:** Rock Island, Illinois


*Death following Vaccination.* By Dr. P. Gregg, of Rock Island, Illinois.

### ARTICLE V.

*Thursday, April 13th.*—Was called to see —— Cheshire, æt. 17 or 18. Found him in the following state: Intense Erysipelatous inflammation occupying one arm from one or two inches below the elbow to axilla, a gangrenous patch two inches square, (the centre of which, was the vaccine puncture,) midway the arm on outer side, and one twice that size, on the under side. Pulse 110 to 120—little resistance on pressure. Tongue moist, slightly furred, and of a blueish shade; delirious, manner hurried, collapsed expression of countenance, picking at the bed clothes, everything indicating rapid sinking of the vital powers. Advised yeast and charcoal poultice, wet with solution of sulph. ferri. Port wine or porter, nitric acid, &c.; 2 P. M., omnia in pejora ruunt; died during the night.

This boy had been vaccinnated on Friday, previous to my seeing him; and, on inquiry, I found that several others had been vaccinnated at the same time, with the same lancet and matter, and in no instance, except this, did any bad consequence follow. Being at a loss to account for the case as it stood, I sent for my partner, Dr. Brackett, who immediately pronounced it a case of the Indiana Epidemic Erysipelas, latent until developed by the vaccine irritation. The family had moved from Kentucky, came through Indiana, staying a few days at Vincennes, where the disease prevailed to some extent; an aunt came to see them, having her head tied up, she was then convalescing from an attack of Erysipelas. A month had elapsed from the time of the boy's exposure to the time of vaccination. Dr. B., who had seen much of the disease in Indiana, deemed this explanation satisfactory as to the origin and cause of the attack.

